# Carbon vs. Titanium Nails in the Treatment of Impending and Pathological Fractures: A Literature Review

**DOI:** 10.3390/jcm13102940

**Published:** 2024-05-16

**Authors:** Elisa Pesare, Cesare Meschini, Matteo Caredda, Federica Messina, Giuseppe Rovere, Giuseppe Solarino, Antonio Ziranu

**Affiliations:** 1 Orthopaedics Unit, Policlinico Universitario di Bari, Department of Translational Biomedicine and Neuroscience ‘DiBraiN’, University of Bari “Aldo Moro”, Piazza G. Cesare 11, 70124 Bari, Italy; giuseppe.solarino@uniba.it; 2 Department of Orthopedics Sciences, Fondazione Policlinico Universitario A. Gemelli IRCCS, 00168 Rome, Italyfederica.messina@unicatt.it (F.M.); antonio.ziranu@unicatt.it (A.Z.); 3 Department of Orthopedics, Ospedale Isola Tiberina-Gemelli Isola, 00186 Rome, Italy

**Keywords:** pathological fracture, carbon, titanium, nail, metastasis

## Abstract

**Background**: Long bones are commonly affected by musculoskeletal tumors, but they also represent one of the most frequent locations for metastases. The treatment is based on pain management and the prevention or stabilization of pathological fractures by intramedullary nailing. While titanium nails are probably the most used, carbon-fiber-reinforced (CFR) nails have emerged as a new option for oncological patients. The aim of this review is to compare titanium and CFR nails according to current findings. **Methods**: Preferred Reporting Items for Systematic Reviews and Meta-analyses (PRISMA) standards were followed: a total of 1004 articles were identified and 10 were included. **Results**: Traditionally, titanium implants are highly valued for their optimal biomechanical properties and ease of insertion, facilitated by their radiopacity. However, the use of titanium poses challenges in radiotherapy due to interference with radiation dosage and the creation of ferromagnetic artifacts. Conversely, CFR implants have emerged as a recommended option for intramedullary fixation, due to their biomechanical and structural properties and their benefits during radiotherapy and follow-up monitoring X-ray. **Conclusions**: CFR nailing represents a promising advancement in the surgical management of oncological patients with long bone metastases. However, further studies are needed to increase surgeons’ confidence in their use.

## 1. Introduction

Pathological fractures of the major long bones have a dramatic physical and psychological impact on patients affected by primary or secondary tumors [1]. Long bones are commonly affected by musculoskeletal tumors, but they also represent one of the most frequent locations for metastatic spread [2]. Indeed, bone ranks as the third most involved site for metastasis, following the lung and the liver [3]. Tumors with a tendency to spread to the bone include those originating from the prostate (32%), breast (22%), and kidney (16%), with additional involvement observed from lung and thyroid cancers (3%) [1]. These four types collectively contribute to 80% of all bone metastases [4]. The most common sites of metastasis, in descending order of frequency, include the spine, pelvis, and long bones (with particular emphasis on the proximal femur) [2].

According to epidemiological research, around 300,000 of the 1.2 million new cancer cases diagnosed in the United States each year will progress to bone metastases [2].

In the case of bone secondary tumors, the treatment is often palliative, with the goals being achieving sufficient pain management and preventing or stabilizing pathological fractures [5]. The prognosis of patients depends on age, performance status, the sites and number of metastases, the free interval of disease, histotypes of the primary tumor, and expected survival [6]. The treatment algorithm for long bone metastases must consider all these factors [7] to select the most effective and appropriate procedure for the patient: this may involve options such as megaprosthesis [8,9] replacement, intramedullary nailing, and minimally invasive surgical techniques such as cementoplasty, ethanol injection, cryoablation, electrochemotherapy, high-intensity radiofrequency ablation, and photodynamic bone stabilization [10].

Recent advancements in surgical techniques have significantly improved the management of this condition. While titanium nails were previously the most commonly implanted, carbon-fiber-reinforced (CFR) nails have emerged as a promising option for oncological patients [11]. CFR nails offer several advantages, including high biocompatibility, favorable biomechanical characteristics, and notably, radiolucency [12]. As a result, CFR nails are being considered a valid alternative to conventional titanium nails, especially for patients scheduled to undergo adjuvant radiotherapy [13]. In fact, adjuvant radiotherapy plays a crucial role in reducing the risk of local progression, and its efficacy hinges on precise target identification and radiation dosage [14]. The use of titanium nails complicates target identification due to interference with radiation dosage and the generation of ferromagnetic artifacts. On the contrary, carbon fiber nails seem to offer a solution by minimizing ferromagnetic artifacts, thereby facilitating effective adjuvant radiotherapy [15].

To date, only a few studies have explored the use of CFR nails in treating bone metastases, often with preliminary findings derived from small sample sizes and short follow-ups. The aim of our review is to compare the utilization of titanium and CFR nails, especially concerning the advantages and disadvantages of their use in oncological patients.

## 2. Materials and Methods

**Figure 1:**
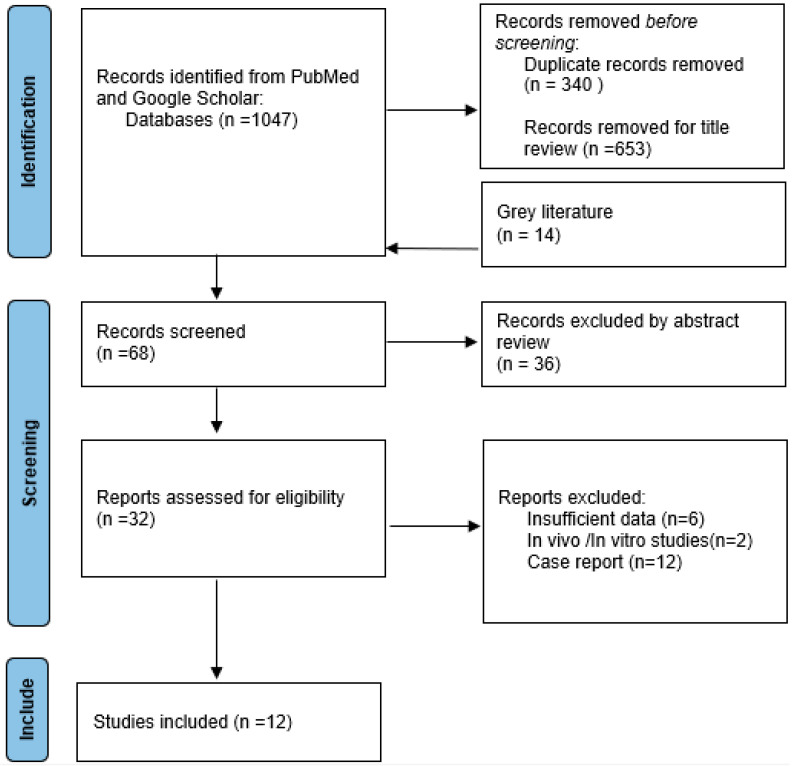
Progression PRISMA flowchart.

The literature review was conducted between January 2000 and March 2024, utilizing a rigorous and systematic methodology in accordance with the Preferred Reporting Items for Systematic Reviews and Meta-analyses (PRISMA) guidelines [16], which is illustrated in the study review progression flowchart (Figure 1).

Keywords including “carbon” and “titanium and “nail” and “oncological” and “pathological fracture” were employed to search the PubMed, Medline, Scopus, and Google Scholar databases, indicating that the search terms were to be found in the title, abstract, and keywords of documents. Logical operators (OR and AND) linked this combination of terms to further restrict the search [17]. Another constraint was imposed on the language of the documents, restricting them to English. Inclusion criteria comprised retrospective and prospective cohorts, and evaluating the use of titanium or carbon-fiber intramedullary nailing for impending or pathological fractures [18]. The final reference list comprised longitudinal studies (both retrospective and prospective) and randomized controlled trials. Exclusion criteria were applied to maintain focus, excluding case reports, expert opinions, prior systematic reviews, letters to editors, and studies not directly related to the review topic.

The grey literature was scrutinized to uncover any additional overlooked research by thoroughly examining the bibliographies of each published study. Through this process, relevant items that might have been missed were identified.

A retrospective investigation was conducted on the selected literature, and pertinent characteristics such as the first author, publication year, and study design were recorded. Information regarding the type of surgery performed, sample size, mean age, tumor type, and mean follow-up period was collected when available.

The screening process involved two authors independently assessing titles and abstracts (P.E. and C.M.), followed by a third author reviewing full-text articles (M.C.). Duplicate publications were eliminated, and any discrepancies were resolved through consultation with a third author experienced in oncological surgery (Z.A.).

## 3. Discussion

At the beginning a total of 1047 articles were identified, while in the end, after the screening process, only 10 papers were selected and included (Table 1).

### 3.1. Pathological and/or Impending Fractures Management

The primary objectives in treating patients with skeletal metastases or bone tumors involve pain alleviation and the restoration of motor function in the shortest time frame possible [4]. Traditionally, this is accomplished through tumor excision and the implantation of endoprostheses or intramedullary fixation [1]. When metastases affect the diaphyses and/or metaphyses of long bones, intramedullary fixation becomes the preferred technique for proper management [2,28].

The surgical goal is to reinforce the affected bone with a definitive, durable, and mechanically stable implant, facilitating pain reduction and enabling early weight-bearing [5]. 

An important differentiation is necessary when discussing the treatment of pathological fractures and impending fractures.

Surgery is warranted when a lesion exceeds an absolute size of >2.5 cm or a relative size of >50% of the cortical defect diameter [29]. 

In all other cases, factors such as the Mirels score [30], tumor staging, life expectancy [31], and sensitivity to adjuvant therapy are taken into account before surgical intervention is considered [4,5,32].

In cases where the prognosis is unfavorable, or when avoiding bed confinement is crucial to permit weight-bearing and prevent local and systemic complications, intramedullary nailing emerges as the preferred surgical approach [14]. 

This method is favored due to its limited surgical exposure and reduced intraoperative bleeding. Additionally, it offers advantages such as minimizing disruption at the tumor site, allowing for the placement of locking screws in a distant, normal bone, enhancing resistance to mechanical and torsional forces, and maintaining the mechanical axis better than a plate [2]. 

The selection of the nail is critical: it should be sufficiently long to protect the entire bone in the event of recurrence or tumor involvement at other sites. It should also have the largest possible diameter and be securely locked proximally and distally with static holes and interlocking screws to control distraction and torsional stresses, thereby facilitating early postoperative function [33,34,35].

### 3.2. Intramedullary Implants

In fracture healing, the biomechanics of the intramedullary implant play a significant role in the healing process, often overshadowing the importance of the biomaterial itself. However, in oncological patients, this concept becomes more complex due to various factors, including the nature of the tumor, its location, and the effects of therapeutic interventions [29].

Traditionally, intramedullary implants have been made from titanium alloy, offering excellent biomechanical properties and ease of insertion, aided by its radiopacity [36].

On the other hand, currently, carbon-fiber-reinforced (CFR) implants present a promising alternative to titanium nails [12]. 

These CFR implants have been suggested for intramedullary fixation in patients with musculoskeletal tumors due to their favorable biomechanical and structural properties. Moreover, they offer advantages during radiotherapy and facilitate fracture monitoring during follow-up [11,12,14].

Carbon fiber nails exhibit low artifact levels, allowing radiotherapists to administer a more effective dose with reduced risks for the patient [25]. This characteristic underscores the potential benefits of CFR implants in the management of oncological patients undergoing radiotherapy [14].

### 3.3. Biomechanical Properties 

Titanium implants possess a notable flexibility and inherent elasticity, allowing them to be significantly over-bent to maintain a curvature [36]. 

This property makes them suitable for patients with compromised health, such as those affected by cancer, as titanium is well tolerated by the body and exhibits excellent biocompatibility [26]. Moreover, titanium implants offer high strength and stability, crucial for providing essential support to bones affected by metastatic lesions or primary bone tumors [36].

On the other hand, CFR implants also boast excellent biomechanical properties, including high strength and stiffness, which are essential for ensuring adequate stability [37]. Carbon fiber, initially discovered in 1860 and utilized for light bulb filaments, has evolved to yield high-performance carbon fibers with exceptional tensile strength and elasticity [15]. In orthopedic applications, carbon fiber exhibits high biocompatibility and chemical inertness, generating minimal cellular toxicity and foreign body reaction [38]. Its elastic modulus closely resembles that of bone, a significant advantage over other implant materials. However, carbon fiber implants lack the flexibility of titanium and cannot be bent or contoured intra-operatively, necessitating precise preoperative planning [38]. 

Additionally, carbon fiber implants demonstrate resilience to fatigue strain, unlike traditional titanium implants that may exhibit higher failure rates, particularly in pathologic fractures [38].

Therefore, while carbon fiber implants offer several advantages, careful consideration and planning are required to mitigate potential risks associated with their use. Traditional implants (titanium) seem to demonstrate higher failure rates, especially in pathologic fractures, often due to nonunion or hardware failure as shown in Ziran et al.’s analysis on the healing process of diaphyseal tibia fractures, where the use of CFR nails led to a more rapid healing process than the use of titanium nails [13,25]. 

In contrast, according to Pala et al., the heightened elasticity of a carbon fiber polymer might permit substantial movement, probably with a higher risk of delayed union and nonunion: their study demonstrated a high incidence of implant failure and nonunion (31% non-union rate) when CFR nails were employed in corrective osteotomies for lower limb deformities [25]. 

### 3.4. Compatibility with Radiotherapy 

In orthopedic oncology, many patients require post-operative radiotherapy [15]. However, traditional titanium implants often generate artifacts that hinder radiation planning, as well as accurate dose calculation and delivery [37].

CFR implants, on the other hand, are compatible with adjuvant radiotherapy, which is a crucial aspect of treatment for oncological patients, although screws used for carbon fiber nail fixation or interlock screws are metallic too, leading to some imaging artifacts [11].

Radiation therapy plays a significant role, particularly in cases involving multiple or painful lesions; in cases of osteolytic lesions, surgical stabilization is often necessary [29]. 

Titanium implants complicate CT-based radiation therapy planning and can have an unpredictable dose-modulating effect during adjuvant radiotherapy [21]. 

The reduced artifact interference provided by CFR implants enables more precise targeting of the radiation beam, minimizing damage to surrounding healthy tissues while effectively treating the tumor. CFR implants also facilitate radiation treatment planning [38].

During radiation therapy, metallic implants affect both surrounding tissues through backscattering and inadvertent dose increase, as well as the lesion to be irradiated due to beam attenuation, compromising the therapeutic effect [21]. In contrast, CFR devices, with their low atomic number and radiation absorption properties similar to surrounding tissues, remain inert to ionizing radiation [11]. 

This characteristic results in minimal disturbance during radiotherapy, allowing for easier and more precise targeting of the tumor.

### 3.5. Reduction in Ferromagnetic Artifacts

Unlike titanium nails, CFR ones do not generate ferromagnetic artifacts, which can interfere with diagnostic imaging techniques. The utilization of carbon fiber implants offers enhanced radiological properties compared to titanium, improving the ease of surveillance imaging and postoperative follow-up [27]. Metallic artifacts often obscure follow-up imaging and hinder the diagnosis of local recurrences [38]. 

This feature ensures that patients with CFR implants can undergo comprehensive imaging studies for accurate diagnosis and follow-up evaluations without interference from artifacts [21]. Additionally, since carbon is non-magnetic, MRI images exhibit higher quality without artifacts, allowing for a better evaluation of pathological tissue [11].

This suggests that CFR-based implants may offer better monitoring of pathological fractures, local recurrence, progression, or response to therapies compared to traditional implants [38].

### 3.6. Radiolucency

One significant advantage of CFR implants lies in their radiolucency. Unlike traditional titanium, CFR implants produce minimal artifact interference on imaging studies such as X-rays and MRIs [24]. This feature enhances the visualization of surrounding tissues and tumor responses to treatment, proving particularly valuable in oncological cases where accurate assessment is crucial [38].

As previously noted, the radiolucency of carbon fiber is advantageous for post-operative imaging studies and follow-up. However, it presents challenges during intraoperative procedures, particularly in confirming the precise placement of implants.

While not necessarily specific to carbon fiber intramedullary nails, an important consideration and potential limitation of carbon fiber implants, in general, is their inability to be contoured compared to other implants, such as those made from titanium [38]. This may require more precise preoperative planning or the use of custom, patient-tailored implants [15].

### 3.7. Clinical Outcomes

**Figure 2:**
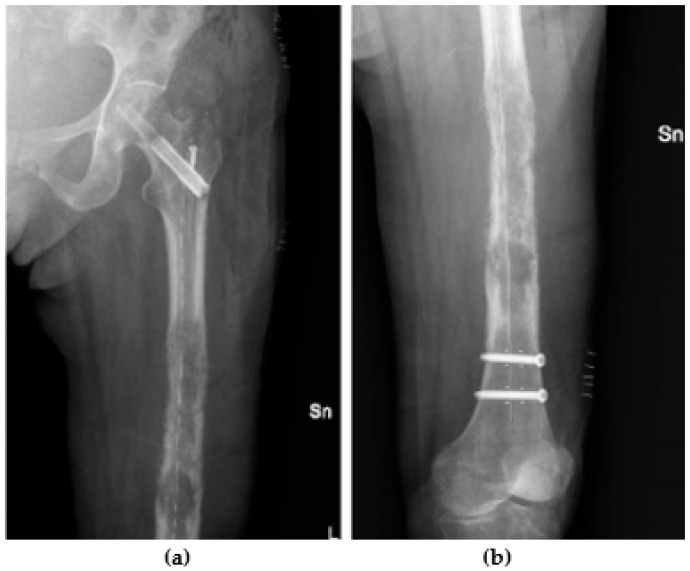
X-ray of a metastatic lesion from pancreatic adenocarcinoma in the left femur in a 74-year-old woman treated with a CFR nail: (**a**) X-ray view of proximal femur; (**b**) X-ray view of distal femur.

While CFR nailing is a relatively newer technique compared to traditional metal implants, emerging clinical studies suggest promising outcomes in terms of pain relief, functional restoration, and overall patient satisfaction [14,15,24] (Figure 2).

However, larger-scale studies with longer follow-up periods are needed to further evaluate the efficacy and long-term outcomes of CFR nailing in oncological patients. Complications in these nails are similar [23].

In Takashima et al.’s report [23], a consolidation of proximal femur fractures treated with CFR nails was observed in 19 out of 20 patients, with no significant complications noted [25]. Piccioli et al. [19] documented two major complications occurring postoperatively in their study: one stress fracture proximal to the distal static screw and one instance of screw loosening. In Pala et al.’s series, two instances of nail breakage were reported in the CFR nail group, with no complications observed in the titanium group [25]. Bhashyam et al. [26] conducted a single-institution retrospective cohort study involving 81 patients treated for humeral diaphyseal bone tumors. They found that CFR humeral intramedullary nails may fail due to tension resulting from bending forces at the distal portion of the bone–cement interface, particularly when large cement spacers are used to replace extensive segments of resected diaphyseal bone, leaving short residual distal bone (5 cm) [26].

Another recent study on 239 patients treated with CFR nails for impending/complete pathological long bone fractures secondary to metastases evaluated incidences of mechanical and nonmechanical complication, with reported comprehensive results comparable with conventional titanium nails rates [27].

### 3.8. Clinical Experience

Titanium nails have been widely utilized in orthopedic surgery for numerous years, boasting a well-established history of safety and effectiveness. Surgeons are accustomed to using titanium implants and have trust in their performance [15].

In some comparative studies available in the current findings, such as Yeung et al. [15], the CFN group exhibited higher estimated blood loss and fluoroscopic time than titanium nails [15]. Probably, the learning curve of surgeons may account in this prolonged surgical time, as the radiolucency of the implants complicates intraoperative techniques. 

Additionally, according to Pala et al. [25], the notable drawbacks of CFN implants, appear to be the increased surgical time and fluoroscopy exposure required.

In their series, the mean duration of surgery was 111 min when using CFR nails. Furthermore, the implantation time of CFR nails was not significantly longer than titanium nails in the femur, and even shorter in the humerus compared to with titanium nails [25]. 

Moreover, in a previous study, intramedullary nailing of the femur in 25 patients for impending or existing pathological fracture fixation also revealed similar surgical parameters, with an average overall operating time of 104 min and a mean blood loss of 744 mL [15,39].

The more recent extensive multicentric study conducted by Piccioli et al. [19], on 53 CFR nails, showed an average surgical procedure time of 69 min and a mean intraoperative fluoroscopy exposure of 96 s.

### 3.9. Availability

While not necessarily a specific issue for carbon fiber intramedullary nails, the production of carbon fiber implants is costly, which could pose a significant barrier to their widespread adoption in orthopedic oncology [38].

In contrast, titanium implants are widely available and routinely utilized in clinical settings, rendering them easily accessible for surgeons treating oncological patients [26].

### 3.10. Limitation

The primary limitation of the review arises from the paucity of studies available on the topic in the current literature. Another challenge lies in their heterogeneity, stemming from the inability to compare populations due to varying oncological diagnoses. Additionally, studies often have short follow-up periods and very small sample sizes. Further investigations are needed to more effectively compare these implants and determine whether the current approach is optimal, or if exploring future prospects would be more beneficial.

## 4. Conclusions

This review represents the first comprehensive comparison between titanium and CFR nailing, specifically in oncological patients, based on the latest research findings available.

In summary, CFR nailing presents a promising advancement in the surgical management of oncological patients with long bone metastases or primary bone tumors compared to traditional titanium implants. Its distinct biomechanical properties, radiolucency, compatibility with radiotherapy, and minimal artifact interference indicate its value in achieving optimal outcomes for oncological populations. However, additional studies may be necessary to increase surgeons’ confidence in the use of CFR implants more extensively.

## Figures and Tables

**Table 1:** Table of included study characteristics: author, title, study type, and year of publication.

Author Name	Title	Characteristics of Included Studies	Year
Capanna et al. [2]	The treatment of metastases in the appendicular skeleton	Review	2001
Zoccali et al. [14]	The CarbofixTM ‘‘Piccolo Proximal femur nail’’: A new perspective for treating proximal femur lesion. A technique report	Clinical trial	2016
Piccioli et al. [19]	Carbon-fiber reinforced intramedullary nailing in musculoskeletal tumor surgery: a national multicentric experience of the Italian Orthopaedic Society (SIOT) Bone Metastasis Study Group	Multicentric study retrospective study	2017
Kojic et al. [20]	Carbon-Fibre-Reinforced PEEK radiolucent intramedullary nail for humeral shaft fracture fixation: technical features and a pilot clinical study	Prospective study	2017
Laux et al. [21]	Carbon fibre/polyether ether ketone (CF/PEEK) implants in orthopaedic oncology	Single-left case series	2018
Sacchetti et al. [22]	Carbon/PEEK nails: a case–control study of 22 cases	Case-control	2019
Takashima et al. [23]	Clinical outcomes of proximal femoral fractures treated with a novel carbon fiber-reinforced polyetheretherketone intramedullary nail.	Clinical trial	2020
Takashima et al. [24]	A carbon fiber-reinforced polyetheretherketone intramedullary nail improves fracture site visibility on postoperative radiographic images	Retrospective study	2021
Pala et al. [25]	Intramedullary nailing for impending or pathologic fracture of the long bone: titanium vs. carbon fiber peek nailing	Prospective case–control study	2022
Yeung et al. [15]	Comparison of carbon fibre and titanium intramedullary nails in orthopaedic oncology	Retrospective case–control study	2022
Bhashyam et al. [26]	Titanium vs. carbon fiber–reinforced intramedullary nailing for humeral bone tumors	Retrospective study	2023
Lozano-Calderon et al. [27]	Outcomes of Long Bones Treated With CarbonFiber Nails for Oncologic Indications: International Multi-institutional Study	Retrospective study	2024

## Data Availability

Not applicable.
